# Excimerlaser-gestützte DALK: Ein Fallbericht aus dem Homburger Keratokonus Center (HKC)

**DOI:** 10.1007/s00347-021-01342-3

**Published:** 2021-02-25

**Authors:** Loay Daas, Loïc Hamon, Navid Ardjomand, Tarek Safi, Berthold Seitz

**Affiliations:** 1 Klinik für Augenheilkunde, Universitätsklinikum des Saarlandes (UKS), Kirrbergerstr. 100, Geb. 22, 66421 Homburg/Saar, Deutschland; 2 Sehzentrum für Augenlaser und Augenchirurgie, Graz, Österreich

**Keywords:** Excimerlaser-gestützte Trepanation, Tiefe anteriore lamelläre Keratoplastik, Prädescemetale Schicht, Dua’s Layer, DALK, Eximer laser-assisted trephination, Deep anterior keratoplasty lamellar keratoplasty, Pre-Descemet’s layer, Dua’s layer, DALK

## Abstract

**Indikationen:**

Ziel der Excimerlaser-gestützten DALK (Excimer-DALK) ist, wie bei der mechanischen DALK, die Behandlung von Keratektasien (Keratokonus und pellucide marginale Degeneration), stromalen Narben oder stromalen Hornhautdystrophien. Voraussetzung für die Operation ist die Abwesenheit von (prä)descemetalen Narben sowie ein gesundes Endothel.

**Operationstechnik:**

Nach der Excimerlaser-gestützten Trepanation auf 80 % der kornealen Dicke an der Trepanationsstelle, einer intrastromalen Lufteingabe (sog. „Big-Bubble“) sowie einer lamellären Hornhautpräparation erfolgt eine lamelläre anteriore Transplantation des endothelfreien Spendergewebes. Diese Technik kombiniert die Vorteile einer DALK und einer Excimerlaser-Trepanation. Wir beschreiben die Schritte einer Excimer-DALK aus unserem Homburger Keratokonus Center (HKC).

**Schlussfolgerungen:**

Die Excimer-DALK stellt bei Patienten mit gutem Endothel eine gute Behandlungsmöglichkeit dar. Bei einer intraoperativen Perforation bleibt die Möglichkeit einer sog. Konversion zur Excimer-PKP mit allen Vorteilen der Excimerlaser-Trepanation.

**Video online:**

Die Online-Version dieses Beitrags (10.1007/s00347-021-01342-3) enthält ein Video.

## Abstract (en)

**Indications:**

The aim of excimer laser-assisted deep anterior lamellar keratoplasty (excimer-DALK) is, as in mechanical DALK, the treatment of keratectasia (keratoconus and pellucid marginal degeneration), stromal scars or stromal corneal dystrophy. A prerequisite for surgery is the absence of (pre‑) Descemet’s scars and an intact endothelium.

**Surgical technique:**

After excimer laser-assisted trephination to 80% of the corneal thickness at the trephination site, intrastromal air injection (so-called big bubble) and lamellar corneal preparation, a lamellar anterior transplantation of the endothelium-free donor tissue is performed. The technique combines the advantages of DALK and excimer laser trephination. We describe the steps of an excimer-DALK from the Homburg Keratoconus Center (HKC).

**Conclusion:**

Excimer-DALK is a viable treatment option for patients with intact endothelium. In cases of intraoperative perforation, conversion to excimer-perforating keratoplasty (PKP) with all the advantages of excimer laser trephination remains feasible.

## Vorbemerkungen

Neben der perforierenden Keratoplastik (PKP) nehmen in Deutschland seit Jahren die lamellären Eingriffe immer mehr Platz in der Hornhautchirurgie ein [6]. Beim Keratokonus ist derzeit die tiefe anteriore lamelläre Keratoplastik („deep anterior lamellar keratoplasty“ [DALK]) eine geeignete chirurgische Behandlung, insbesondere bei jungen Patienten mit erhöhtem Risiko einer Immunreaktion des Transplantates, wie z. B. bei Neurodermitis [4]. Die erste DALK wurde 1959 von Hallerman durchgeführt [8]. Die Operationstechnik wurde danach – unter anderem infolge der Einführung der intraoperativen Tomographie – mehrmals modifiziert. Allerdings besteht zurzeit noch keine standardisierte Methode. Wir stellen den Fall einer Excimerlaser-gestützten DALK (Excimer-DALK) dar.

## Definition und Operationsindikation

Die Excimer-DALK besteht aus einer Excimerlaser-gestützten Trepanation (auf 80% der mit dem Vorderabschnitts-OCT gemessenen mittelperipheren Hornhautdicke) mit darauffolgender Exzision der oberflächlichen Stromalamelle und Injektion von Luft ins verbliebene tiefe Stroma zur Separation von Descemet-Membran und posteriorem Stroma. Dann wird – erst nach gelungener Dissektion – eine anteriore Spenderlamelle gleicher Abmessung transplantiert, von der die Descemet-Membran zuvor abpräpariert wurde. Die Spenderlamelle wird mit 2 fortlaufenden Kreuzstichnähten nach Hoffmann an der Wirtshornhaut befestigt. Ziel der Excimerlaser-Trepanation wie bei der Excimerlaser-gestützten PKP (Excimer-PKP) [12, 13] ist eine optimale Visuserholung mit niedrigem postoperativem Astigmatismus. Die postoperativen Ergebnisse der DALK sind grundsätzlich ähnlich zur PKP, wie bereits für die mechanische Trepanation gezeigt wurde [2]. Der Hauptvorteil der DALK im Vergleich zur PKP ist die Minimierung der Gefahr einer Immunreaktion des Transplantats bei Belassen des eigenen Endothels [11]. Der Nachteil im Vergleich zur PKP ist der zeitliche Aufwand, die nicht standardisierte, erfahrungsabhängige Methode und die höhere Rate einer Fadenlockerung (im Vergleich zur PKP) [3]. Die Indikationen und Kontraindikationen einer Excimer-DALK sind ähnlich einer mechanischen DALK. Die häufigsten Indikationen für eine DALK sind die Keratektasien (Keratokonus und pellucide marginale Degeneration). Andere, seltenere Indikationen sind stromale Narben oder stromale Hornhautdystrophien. Voraussetzung für die Operation ist die Abwesenheit von (prä-)descemetalen Narben sowie ein gesundes Endothel. Die absolute Kontraindikation einer DALK ist eine Beteiligung des Endothels: endotheliale Narben, eine endotheliale Dystrophie oder eine eingeschränkte Endothelzellzahl. Bei Zustand nach akutem Keratokonus kann nur eine PKP durchgeführt werden. Darüber hinaus sollte bei Herpeskeratitis keine DALK durchgeführt werden aufgrund der möglichen Herpesreaktivierung im eigenen Endothel [9].

## Vorbereitung

Zur Vermeidung einer postoperativen zweiten Vorderkammerbildung, im Fall einer intraoperativen Mikroperforation und bei fehlender intraoperativer optischer Kohärenztomographie (iOCT) empfehlen wir die Injektion von Luft/Gas in die Vorderkammer nach den 2 fortlaufenden Kreuzstichnähten nach Hoffmann. Aus diesem Grund ist eine präoperative große Nd:YAG-Iridotomie von ca. 1 mm bei 6 h als Prophylaxe einer postoperativen Luftblocksituation mit Tensioentgleisung geboten.

## Anästhesie und Lagerung

Die Anästhesie erfolgt in der Regel in Intubationsnarkose (ITN). Der Patientenkopf wird in einer Kopfschale horizontal gelagert.

## Fallbeschreibung und Operationstechnik

Ein 44-jähriger Mann stellte sich mit einem bestkorrigierten Visus von 0,3 (nach Snellen) am rechten Auge vor. Bei ihm wurde ein Keratokonus Stadium A3B4C2D2- (nach Belin) diagnostiziert [7]. Anamnestisch gab der Patient eine Kontaktlinsenunverträglichkeit an. Eine Excimer-DALK wurde durchgeführt. Die unterschiedlichen Schritte dieser Operation sind im Video 1 dargestellt.

**Figure 1:**
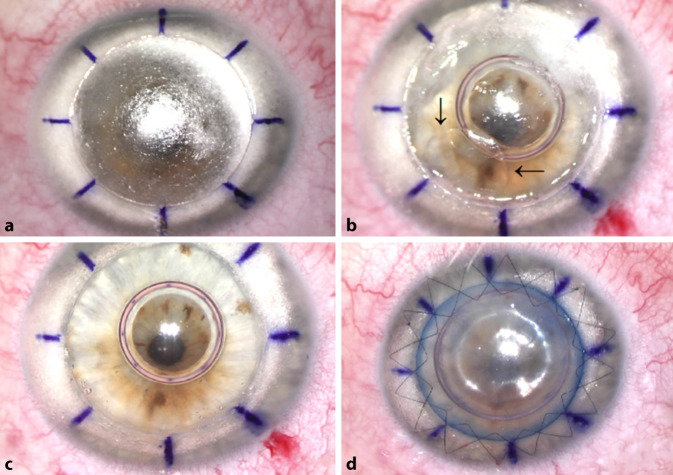

Die Operation beginnt mit einer Excimerlaser-gestützten Trepanation auf 80% der mit dem Vorderabschnitts-OCT gemessenen mittelperipheren Hornhautdicke. Nach Exzision der anterioren Stromalamelle („Debulking“) wurde eine intrastromale Luftinjektion zur Separation von Stroma und Descemet-Membran mittels einer 30-Gauge-Nadel nach der „Big Bubble“-Technik von Anwar [1] durchgeführt, die zur Bildung einer „Big Bubble“ vom Typ 1 zwischen dem Stroma und der prädescemetalen Schicht („Dua’s layer“) [5] führte (Abb. 1a). Eine andere Luftblase wurde in die Vorderkammer via Parazentese gegeben. Diese Luftblase in der Vorderkammer spielt insbesondere bei nicht tomographisch assistierter Operation eine wichtige Rolle zur optischen Sicherstellung, dass die „Big Bubble“ in der richtigen Hornhautschicht (zwischen posteriorem Stroma und prädescemetaler Schicht bei Typ 1 und zwischen prädescemetaler Schicht und Descemet-Membran bei Typ 2) liegt. Dies erkennt man daran, dass sich die Luftblase auf die Seite verschiebt und nierenförmig imponiert. Nach Injektion der „Big Bubble“ und der Luftblase in die Vorderkammer wurde das restliche Stroma entfernt. Zu diesem Zeitpunkt wurde durch die teilweise klare Hornhaut eine „Big Bubble“ vom Typ 2 zwischen der prädescemetalen Schicht und der Descemet-Membran bemerkt (Abb. 1b – Pfeile). Die Entscheidung wurde getroffen, die „Big Bubble“ vom Typ 2 vorsichtig zu „entlüften“ und die prädescemetale Schicht zu durchtrennen und zu exzidieren, um einen früheren und besseren postoperativen Visus aufgrund geringerer residualer Hornhautrübung („Interface-Problematik“) zu erreichen, wobei das hohe Perforationsrisiko ebenfalls berücksichtigt wurde. Schließlich sah man auf die kristallklare Descemet-Membran (Abb. 1c). Nach Dissektion des Empfängerstromas und der prädescemetalen Schicht erfolgte bei der Spenderlamelle, die vorher auch mit dem Excimerlaser identisch wie für eine Excimer-PKP trepaniert wurde, eine manuelle Descemetorhexis nach Trypanblau-Färbung. Das Transplantat wurde – nach 8 temporären Situationsnähten – mittels zweier fortlaufender Kreuzstichnähte nach Hoffmann im Empfängerbett fixiert (Abb. 1d).

## Besonderheiten der Operationstechnik

Die Besonderheit der Technik der Excimer-DALK im Vergleich zur „mechanischen“ DALK ist die Trepanation der Empfänger- und Spenderhornhaut mittels Excimerlaser. Wie bereits bei der Excimer-PKP gezeigt, führt die Excimerlaser-Trepanation zu einem signifikant reduzierten postoperativen Astigmatismus nach Fadenentfernung [12–14]. Dies sollte folglich auch bei Excimer-DALK gelten. Weitere Studien sind nichtsdestoweniger notwendig, um diese Hypothese zu beweisen. Verschiedene Studien zeigten bei einer mechanischen DALK keinen Unterschied in der Schnelligkeit und Entwicklung der Visuserholung im Vergleich zur PKP [2, 10, 12]. Unseres Wissens ist ein Vergleich der Visuserholung zwischen Excimer-DALK und Excimer-PKP noch nicht untersucht worden. Bei intraoperativer Perforation der Descemet-Membran muss von einer DALK üblicherweise auf eine PKP umgestiegen werden (sog. Konversion). Die Empfänger- und Spenderhornhaut sind in diesem Fall mit dem Excimerlaser in typischer Weise vorbereitet. Damit profitiert der Patient von den Vorteilen einer Excimerlaser-Trepanation auch bei einer Konversion zur PKP.

## Postoperative Behandlung und Verlauf

Die Nachsorge ähnelt der Nachsorge nach einer PKP. Obwohl es nicht zu endothelialen Immunreaktionen kommen kann, wird zur Vermeidung von epithelialer und stromaler Immunreaktion ein Steroidschema empfohlen. Die Steroidtherapie beginnt mit Prednisolonacetat-Augentropfen 5‑mal am Tag sowie systemisch Methylprednisolon mit Reduktionsschema über 2 Wochen. Sechs Monate postoperativ betrug der bestkorrigierte Visus mit liegenden Hornhautfäden 0,5 (nach Snellen) mit einem regulären kornealen Astigmatismus von 5 dpt.

## Fehler, Gefahren, Komplikationen

Bei einer Perforation oder einer unzureichenden Freilegung der Descemet-Membran sollte die Operation zu einer Excimer-PKP konvertiert werden.

## Fazit für die Praxis

- Bei Excimer-DALK erfolgt die Excimerlaser-Trepanation analog zur Excimer-PKP, ist aber auf 80 % der kornealen Dicke beschränkt.
- Eine Exzision der prädescemetalen Schicht („Dua’s layer“) ist möglich unter Berücksichtigung des Perforationsrisikos.
- Bei intraoperativer Perforation der Descemet-Membran ist eine Konversion zur Excimer-PKP mit allen Vorteilen der Excimerlaser-Trepanation möglich.

## Supplementary Information




